# Alterations in VASP phosphorylation and profilin1 and cofilin1 expression in hyperoxic lung injury and BPD

**DOI:** 10.1186/s12931-018-0938-1

**Published:** 2018-11-21

**Authors:** Mehboob Ali, Kathryn Heyob, Trent E. Tipple, Gloria S. Pryhuber, Lynette K. Rogers

**Affiliations:** 1 Center for Perinatal Research, The Research Institute at Nationwide Children’s Hospital, 575 Children’s Cross Road, Columbus, OH USA; 2 Department of Pediatrics, University of Alabama at Birmingham, Birmingham, AL USA; 3 Department of Pediatrics, University of Rochester Medical Center, Rochester, NY USA; 4 Department of Pediatrics, The Ohio State University, Columbus, OH USA

**Keywords:** Hyperoxia, BPD, Actin binding proteins, L-MSCs, CD146, VASP, VASP^pS157^, VASP^pS239^, Profilin1, Cofilin1

## Abstract

**Background:**

Hyperoxia is a frequently employed therapy for prematurely born infants, induces lung injury and contributes to development of bronchopulmonary dysplasia (BPD). BPD is characterized by decreased cellular proliferation, cellular migration, and failure of injury repair systems. Actin binding proteins (ABPs) such as VASP, cofilin1, and profilin1 regulate cell proliferation and migration via modulation of actin dynamics. Lung mesenchymal stem cells (L-MSCs) initiate repair processes by proliferating, migrating, and localizing to sites of injury. These processes have not been extensively explored in hyperoxia induced lung injury and repair.

**Methods:**

ABPs and CD146^+^ L-MSCs were analyzed by immunofluorescence in human lung autopsy tissues from infants with and without BPD and by western blot in lung tissue homogenates obtained from our murine model of newborn hyperoxic lung injury.

**Results:**

Decreased F-actin content, ratio of VASP^pS157/VASPpS239^, and profilin 1 expression were observed in human lung tissues but this same pattern was not observed in lungs from hyperoxia-exposed newborn mice. Increases in cofilin1 expression were observed in both human and mouse tissues at 7d indicating a dysregulation in actin dynamics which may be related to altered growth. CD146 levels were elevated in human and newborn mice tissues (7d).

**Conclusion:**

Altered phosphorylation of VASP and expression of profilin 1 and cofilin 1 in human tissues indicate that the pathophysiology of BPD involves dysregulation of actin binding proteins. Lack of similar changes in a mouse model of hyperoxia exposure imply that disruption in actin binding protein expression may be linked to interventions or morbidities other than hyperoxia alone.

## Background

Vasodilator-stimulated phosphoprotein (VASP) is one of the three mammalian Ena/VASP family members that link the cell membrane, signal transduction pathways, and the actin cytoskeleton. VASP activity is regulated by phosphorylation at serine 157 (VASP^pS157^) or 239 (VASP^pS239^), respectively [1–3](reviewed in [4]). VASP interacts with other actin binding proteins (ABP) such as profilin1 to promote actin polymerization [5–7] and cofilin1, to sever F-actin into ADP-actin monomers, depolymerizing F-actin and facilitating actin stabilization [5, 8–10]. Other investigations have identified differential VASP phosphorylation and its association with profilin1 and/or cofilin1 to regulate vascular remodeling and vasodilation/vasoconstriction in pulmonary vascular morbidities [11, 12] and in changes in endothelial permeability in the context of lung injury [13]. Additionally, in a lipopolysaccharide (LPS) induced acute lung injury (ALI) animal model, VASP knockout mice demonstrated greater pulmonary injury when compared to wild type animals due to decreased alveolar capillary barrier properties [14]. Moreover, airway smooth muscle cells and migrating vascular endothelial cells require VASP^pS157^ for membrane or leading edge localization [15].

Improved understanding of newborn lung development and pathophysiology has led to improved neonatal care over the last 20 years [16]. However, elevated oxygen tensions are still used to treat infants with Respiratory Distress Syndrome and contributes to the development of Bronchopulmonary Dysplasia (BPD) [17, 18]. Preterm infants with BPD display deficits in alveolarization, pulmonary vascular development, cellular proliferation, and suppressed injury repair [18, 19]. In response to lung injury, lung mesenchymal stem cells (L-MSCs) migrate to injury sites and accelerate repair processes [20–23]. MSCs have been investigated for their therapeutic potential in preterm infants with chronic lung disease yet the results have been mixed and concern for safety has prevented clinical trials (reviewed in [24]). Migration and proliferation of L-MSCs are likely to be regulated by alternative phosphorylation of actin binding proteins (ABPs). Moreover, MSCs can secrete nitric oxide (NO) or S-nitrosothiols (SNOs), which regulate vasculogenesis via the soluble guanyl cyclase C mediated cGMP/PKG pathway which in turn could regulate VASP phosphorylation [25, 26]. A recent clinical study found an association between increased cGMP, VASP phosphorylation at serine 239, and improved lung function in preterm infants with early symptoms of BPD, indicating a possible involvement of VASP [27].

In the present study, we utilized human neonatal lung autopsy tissue and our murine newborn hyperoxia exposure model to test the hypothesis that changes in VASP phosphorylation state and expression ABPs profilin1 and cofilin1 correlate with hyperoxic lung injury and repair and that changes in these ABPs are associated with recruitment of MSCs to the injured lung.

## Materials and methods

### Human lung tissues F-actin content

Human lung autopsy tissues from infants that died with BPD and infants that died at similar postnatal ages from non-pulmonary causes (control) were obtained from Dr. Gloria Pryhuber and collected as part of the BRINDL repository and Lung Map consortium [28]. Study inclusion/exlusion criteria, IRB approval and parental consent has been previously described [29]. Lung tissue sections were obtained from 16 infants, 8 infants that died with BPD and 8 infants that died of other non-pulmonary causes, (*n* = 8 in each group) and the clinical descriptions and co-morbidities of the infants from whom these samples were collected have been previously published [29]. F-actin content in tissue was estimated by using Alexafluor-633 tagged phalloidin (Invitrogen, Eugene, Oregon) and DAPI (Invitrogen, Carlsbad, CA) for the nucleus. Images were acquired in a blinded fashion using a confocal microscope (LSM510, Carl Zeiss, Jena, Germany) with 40X objective. Four photomicrographs were obtained from each tissue section assessed independently and averaged for each individual thus creating an *n* = 8 for each diagnosis. Identical confocal settings were applied to acquire all images across conditions. F-actin content/cell was quantified using NIH Image J analysis.

### Human lung autopsy tissues ABPs analysis

Tissues were processed for immunofluorescence (IF) labelling using standard protocols [30]. Tissue sections were stained with primary anti-human polyclonal antibodies targeting profilin1 (catalog# 3237), cofilin1(catalog# 5175), VASP (catalog# 3112), VASP^pS239^ (catalog# 3114) (Cell Signaling Technology, Inc., Danvers, MA), VASP^pS157^ (catalog# sc-23,506-R) (Santa Cruz, Dallas, Texas) (dilution, 1:500), followed by secondary antibodies Alexa 488 labeled IgG (1:1000) and nuclei stained with DAPI (Invitrogen, Carlsbad, CA). Images were taken using Carl Zeiss’s Axio Scope A1 Polarized Light Fluorescent Microscope (Carl Zeiss, Jena, Germany) with 40X magnification and identical settings. Four photomicrographs were obtained from each tissue section assessed independently and averaged for each individual thus creating an *n* = 8 for each diagnosis. Intensity of image color was quantified using NIH image J software.

### Newborn hyperoxia exposure

Animal study protocols were approved by the IACUC at The Research Institute at Nationwide Children’s Hospital and pregnant dams were dated as to time of delivery. C3H/HeN mice were bred and at least 2 dams were required to deliver within 12 h. Once born, the pups were randomized and equally distributed between the two dams. One dam and litter was placed in a plexiglass chamber containing 8 L/min flow of 85% O_2_ while the corresponding dam and litter remained in room air (RA). The dams were switched every 24 h to prevent oxygen toxicity. The pups were removed from oxygen exposure on day 14 and allowed to recover in room air. On day of life 7 or 56 the pups were euthanized and lung tissues were snap-frozen in liquid N_2_. At term, newborn mice are in the saccular stage of lung development much like a 26–36 week gestation human infant [31, 32]. Day 7 was chosen to represent acute lung injury during the course of exposure and at the time point when the inflammatory responses are greatest and alveolarization is actively taking place [33] and day 56 was chosen as point after recovery to identify any permanent changes in protein expression. Tissues from 4 mice, one from each of 4 independent litters, were used for further analyses.

### Mouse lung homogenate ABPs analysis

Snap-frozen lung tissues were homogenized (10% *w*/*v*) in solution of 0.1 M Na phosphate buffer containing 5 mM EDTA (pH 7.4) using TissueLyser II (Qiagen, Germantown, MD) and centrifuged at 12000 rpm for 20 min to prepare post mitochondrial supernatants. Protein concentrations were measured using Bio-Rad protein reagent (Bio-Rad, Hercules, CA). Equal amounts of post mitochondrial supernatants were dissolved in SDS sample buffer and proteins were separated by SDS-PAGE, transferred to nitrocellulose membranes, and probed with anti-human polyclonal antibodies targeting profilin1 (catalog# 3237), cofilin1(catalog# 5175), VASP (catalog# 3112), VASP^pS239^ (catalog# 3114) (Cell Signaling Technology, Inc., Danvers, MA), VASP^pS157^ (catalog# sc-23,506-R) (Santa Cruz, Dallas, Texas) (dilution, 1:500), and rabbit monoclonal antibodies targeting GAPDH (D16H11) (catalog #5174)(1:10,000) (Cell Signaling Technology, Inc., Danvers, MA). Finally, membranes were probed with horseradish peroxidase (HRP)-conjugated secondary antibody (dilution, 1:1000) (BD Pharminogen®, Franklin lakes, NJ) for 1 h at room temperature and specific bands were visualized employing Amersham™ECL™ Prime Western Blotting Detection Reagent (GE Healthcare, Buckinghamshire, UK) and the band intensity was measured by densitometry.

### L-MSCs marker (CD146) analysis in human lung autopsy tissues and mouse lung homogenate

CD146 expression and levels in human lung autopsy tissues and mouse lung homogenate was assessed by IF staining and western blot analyses respectively. Human MCAM/CD146 Ab (R&D Systems, Minneapolis, MN) was used as primary antibody while HRP and Alexa 488 labeled IgG (1:1000) were used as secondary antibodies for western and IF analysis, respectively. Nuclei in lung tissue biopsy sections were stained with DAPI (Invitrogen, Carlsbad, CA) and 40X magnification images (4 from each slide) were obtained and analyzed as described previously.

#### Statistics

Human lung tissues (*n* = 8, each group) and mouse lung homogenate (*n* = 4, each group) were considered in present study. Statistical analyses were performed using either two-way ANOVA or Welch’s unequal variances t-test (GraphPad Prism, version 7.03; GraphPad Software, Inc.) and significance is presented as “p” value or for post hoc analyses is indicated as *, *p* < 0.05.

### Result

### Lung tissue F-actin contents were lower in infants who die with BPD

**Fig. 1:**
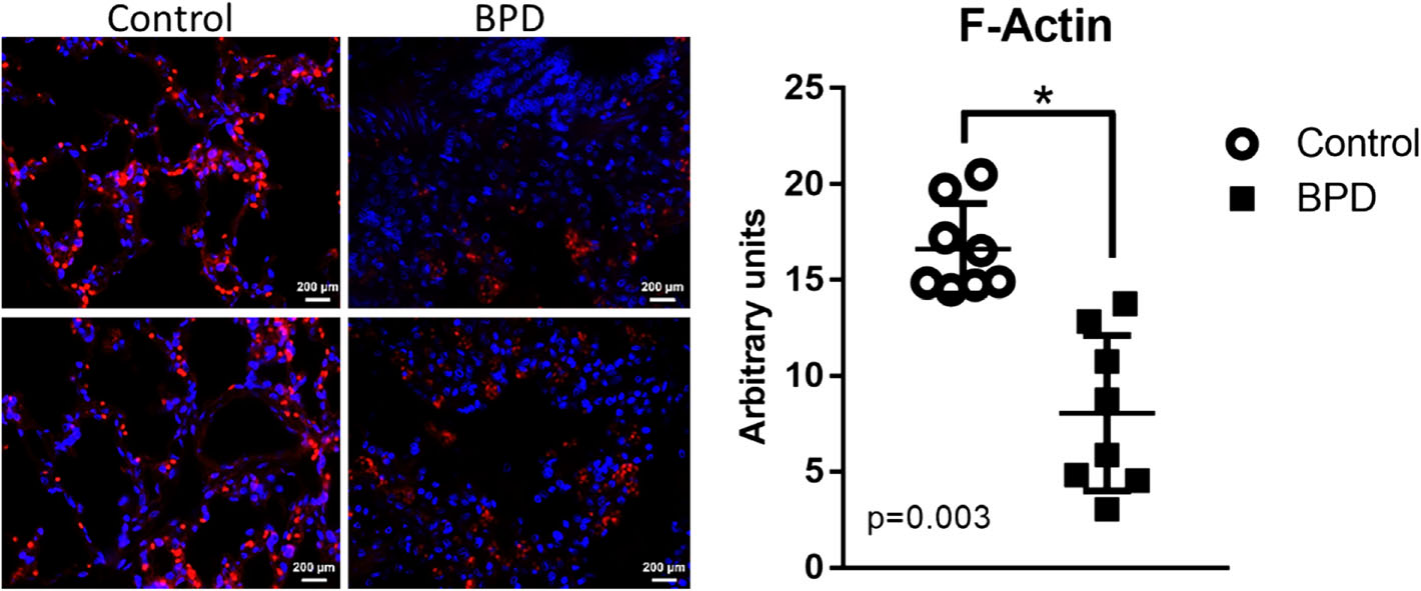
Human lung autopsy tissues F-actin content. Lung tissues sections were obtained from infants that died with BPD (closed squares) or infants that died at similar ages from non-pulmonary illnesses (open circles). Phalloidin used to stain F-actin in red while nuclei stained with DAPI in blue. Confocal microscopy images at 40X magnification processed in NIH Image-J software to measure F-actin contents. Four independent images were obtained from each slide and averaged for each individual creating *n* = 8 for each group. Statistical analysis was performed using Welch’s unequal variances t-test, and significance is indicated as *, *p* < 0.05; compared to controls

F-actin contents were assessed by immunofluorescence in human lung autopsy tissues from infants that died with BPD and infants that died at similar postnatal ages from non-pulmonary causes (control). Lower F-actin content (Fig. 1), was observed in the tissues of infants that died with BPD than in the control group. These differences may represent an overall suppression of actin dynamics and/or cell growth.

### Changes in VASP phosphorylation, and profilin 1 and cofilin 1 expression were observed in infants who died with BPD

**Fig. 2:**
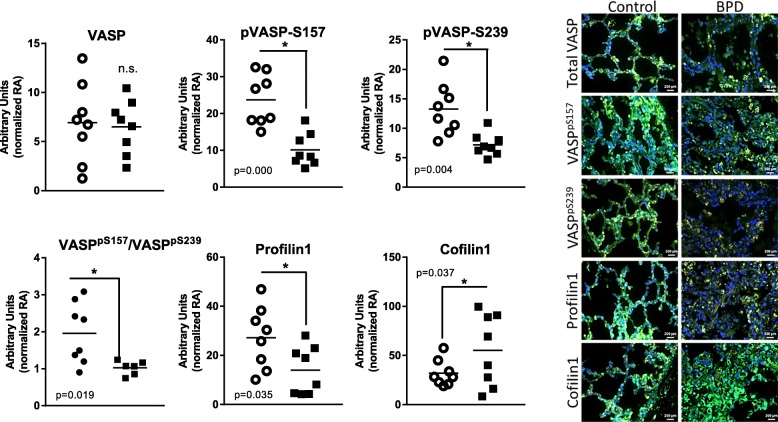
VASP phosphorylation, profilin1 and cofilin1 expression in human lung autopsy tissue. Lung tissues sections were obtained from infants that died with BPD (closed squares) or infants that died at similar ages from non-pulmonary illnesses (open circles). Fluorescent microscopy followed by NIH Image J analysis was used to quantify VASP expression and differential phosphorylation, and profilin1 and cofilin1 expression in human autopsy samples. Images are 40X magnification. Green, VASP, phosphor-VASP, profilin1 and cofilin1 stained with specific antibody against each protein; red, F-actin stained with phalloidin; and blue, nuclei stained with DAPI. Four independent images were obtained from each slide and averaged for each individual creating *n* = 8 for each group. Statistical analysis was performed using Welch’s unequal variances t-test, n = 8 in each group, and significance is indicated as *, *p* < 0.05; and n.s. = non significant compared to respective controls

Cofilin1, profilin1 and VASP phosphorylation were assessed by immunofluorescence. Our data did not indicate differences in total VASP fluorescence; however, we did detect lower levels of VASP phosphorylation in tissues from infants who died with BPD which created a net decrease in the ratio of VASP^pS157^/VASP^pS239^ (Fig. 2). Profilin1 immunofluorescence was lower and cofilin 1 immunofluorescence was greater in tissue sections from infants who died with BPD when compared to infants who died of non-respiratory causes (Fig. 2).

### VASP phosphorylation, and profilin 1 and cofilin 1 levels in lung tissues homogenates from mouse pups exposed to hyperoxia

**Fig. 3:**
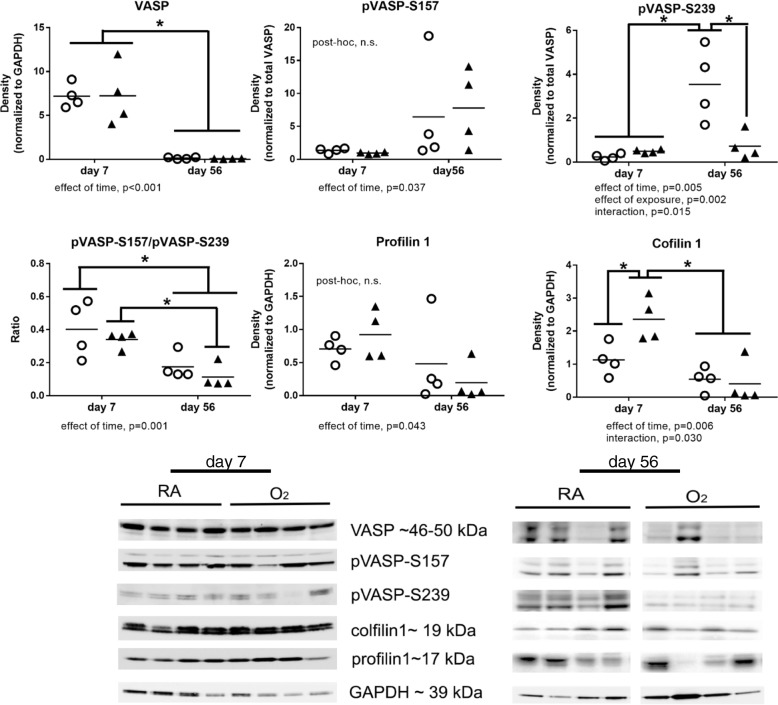
VASP phosphorylation, profilin1 and cofilin1 level in lung tissue homogenates from mouse pups exposed to hyperoxia until day 7. Western blot analysis was performed on whole lung tissue homogenates obtained from mice exposed to room air (RA, open circles) or 85% O_2_ (O_2_, closed triangles) from birth through day 7 or at day 56 after 14 days of hyperoxia exposure and 42 days of room air recovery. Band intensity was quantified by densitometry and statistical analysis was performed using two-way ANOVA with Tukey’s post hoc, *n* = 4 in each group. *, *p* < 0.05 and n.s. = non significant

Western blot analyses of lung tissue homogenates from mouse pups exposed to hyperoxia for 7 days demonstrated greater expression cofilin 1 than in lungs from room air exposed controls (Fig. 3). At 56 d, VASP^pS239^ protein levels were lower in lungs from mice previously exposed to hyperoxia than in mice raised in room air from birth (Fig. 1). There was a time dependent decrease in total VASP, profilin 1, and cofilin 1 between day 7 and day 56 indicating that these proteins were likely involved in lung growth and development. Alternatively, there was a time dependent increase in VASP^pS157^ in both the room air and hyperoxia groups and an increase in VASP^pS239^ in the room air group that was blunted by the previous hyperoxia exposure. The time dependent changes in VASP phosphorylation altered the relative ratio of VASP^pS157^/ VASP^pS239^ which was overall lower at day 56.

### L-MSCs (CD146) in mouse lung homogenates and human lung autopsy tissues

**Fig. 4:**
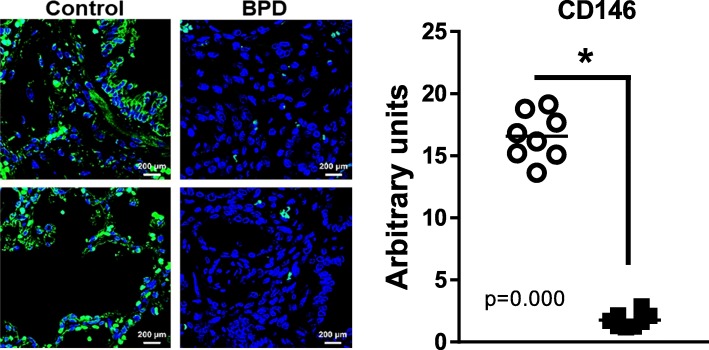
L-MSCs (CD146) levels in lung tissue homogenates from mouse pups exposed to hyperoxia. Western blot analysis was performed on whole lung tissue homogenates obtained from mice exposed to room air (RA, open circles) or 85% O_2_ (O_2_, closed triangles) at day 7 and 56. Band intensity was quantified by densitometry and statistical analysis was performed using two = way ANOVA with Tukey’s post-hoc. *, *p* < 0.05 and n.s. = non significant

**Fig. 5:**
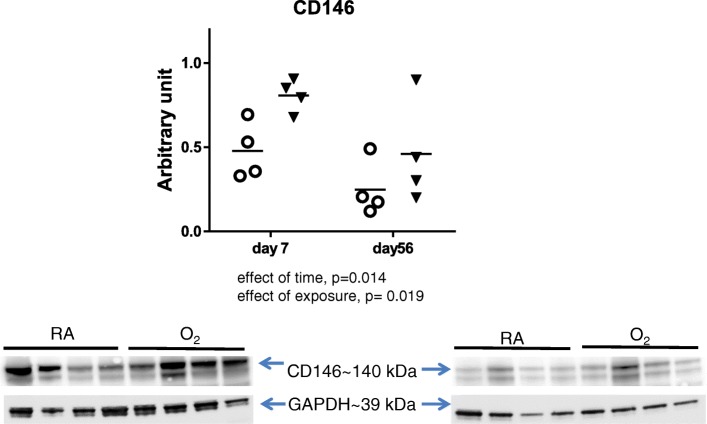
L-MSCs marker (CD146) expression in human lung autopsy tissues. Lung tissues sections were obtained from infants that died with BPD (closed squares) or infants that died at similar ages from non-pulmonary illnesses (open circles). Green, CD146 stained with specific antibody; and blue, nuclei stained with DAPI. Confocal microscopy images at 40X magnification processed in NIH Image-J software. Statistical analysis was performed using Welch’s unequal variances t-test, *n* = 8 in each group, and significance is indicated as ***, *p* < 0.0001; compared to controls

CD146 expression was measured by immunofluorescence in lung autopsy tissues and by western blot in mouse lung homogenates. Lower levels of CD146 expression were detected in lung autopsy tissues obtained from infants who died with BPD compared to non-BPD controls (Fig. 4). CD146 levels in murine lungs tended to be higher in hyperoxia-exposed mice than in room air controls (Fig. 5) as there was an effect of both time and exposure (two-way ANOVA). The western blots (specifically in Fig. 5) possessed some small inconsistencies such as minor bubbles but these were outside the quantification area and did not affect the results as a whole and given the nature of these samples would have been difficult to repeat. We also observed double bands for GAPDH on the day 7 blot, however this is consistent with many other publications of this protein [34–36] and both bands were quantified by densitometry and used for normalizing the western blot.

## Discussion

The relative ratio of VASP^pS157^ to VASP^pS239^ represents the activity of VASP to add actin monomers and promote growth and migration. In the present study, we investigated whether alternative phosphorylation of VASP or other ABPs may be associated with the deficit in lung growth and repair observed in infants that develop BPD.

To accomplish our goals, we obtained human autopsy tissues from Dr. Gloria Pryhuber for analysis and employed our murine C3H/HeN model of neonatal hyperoxia exposure [37, 38]. In human lung autopsy tissues obtained from infants who died with BPD and infants that died at similar ages with non-pulmonary morbidities (for demographic data see reference [29]), we observed less total F-actin content than in tissues from infants who died of BPD than controls (Fig. 1). This observation may be due to decreased alveolarization which, in turn, provided less tissue to analyze per microscope field or may represent a decrease in lung growth and development. Using immunofluorescence (IF) labelling, the contents of VASP, VASP^pS157^, VASP^pS239^, profilin1, and cofilin1 were assessed. Our data indicated overall lower levels of protein containing either sites of VASP phosphorylation and a decrease in VASP^pS157/VASPpS239^ ratio in the BPD tissues compared to controls. This data indicated either lower VASP^pS157^ expression and/or increased VASP^pS239^ expression in infants with BPD than in control tissues (Fig. 2). Both of these interpretations could implicate an impaired tissue or cellular repair mechanism. Lower profilin1 and elevated cofilin1 levels could also be interpreted as impaired growth mechanisms as they would contribute to a decreased availability of actin monomers and increased actin end-capping.

To identity whether the changes observed in the autopsy tissues were due to acute hyperoxia exposure or to later adaptive responses, we analyzed changes in ABPs at 7d of continuous hyperoxia and at 56d, well after recovery. At 7d there were no changes in VASP expression or phosphorylation or profilin 1 expression (Fig. 3). We did however identify an increase in cofilin 1 expression in the hyperoxia-exposed mice compared to controls. At 56d of life, VASP^pS239^ was lower in mice previously exposed to hyperoxia than in RA controls (Fig. 3). Expression of total VASP, VASP^pS157^, profilin 1 and cofilin 1 were higher at day 7 than at day 56 and this decrease likely coincides with completion of lung development prior to day 56. Interestingly, VASP^pS239^ is increased at the later time point and this increase is prevented in the mice previously exposed to hyperoxia. The relevance of this increase is uncertain but may reflect a maintenance or homeostatic effect of this particular modification that is prevented by the early exposure to hyperoxia and may reflect a later life susceptibility to disease.

Collectively, these data indicate that while VASP proteins and phosphorylation are likely involved in the pathology of human BPD, their dysregulation it is not likely due to hyperoxia exposure alone as in our mouse model but rather other stressors such as mechanical ventilation or potentially surfactant insufficiency. Cofilin1 levels were increased in both human tissues and 7d mouse tissues. Cofilin1 is highly regulated during development as it spatially and temporally regulates cell morphology and morphogenesis in developing tissues [7]. The aberrant increases observed in the human tissues as well as our mouse model could contribute to poor lung growth and decreases in alveolarization and should be a focus of further investigation.

Repair processes such as those initiated by hyperoxic injury, instigate proliferation of L-MSCs and migration to the site of injury [21, 23, 24, 39]. Characterization of multipotent stem cells and L-MSCs indicated that CD146 is a marker of MSCs [40, 41]. As a marker of tissue repair, we used CD146 expression in human autopsy lung tissue samples and lung tissue homogenates from our mouse model of hyperoxia injury to identify the relative amount and the localization of MSCs. We observed decreased CD146+ L-MSC expression in BPD tissues compared to control (Fig. 4) suggesting that there are fewer L-MSCs to facilitate growth and repair function. Alternatively, we observed increased CD146 expression at both day 7 and day 56 in the mouse tissue homogenates (Fig. 5). In the human tissues, injury and disease has progressed to a state that death occurred and it is likely that repair mechanisms were no longer active or had been overwhelmed by the extent of injury. At both time points in the mouse model, repair and regeneration was still taking place and the increase in L-MSCs would corroborate the role of these cells types in repair. Overall our data supports the previous findings that induction of L-MSC-mediated repair takes place in response to hyperoxia and persists but that there reaches a point in injury where repair mechanisms are no longer sufficient. Several differences were observed between mouse and human tissue measurements and these may be attributable to numerous factors. In the mouse, we measured these parameters either during acute injury (at 7d of exposure) or very late after recovery (at 56 d). In the infant lungs, there was no recovery and injury had progressed much further than observed with only 14d of hyperoxia exposure as in the mice. There are also many other confounding factors in the human tissues. For example, BPD did not exist as a single morbidity but was one of many as indicated in our previous publication [29]. Exposure to mechanical ventilation and excessive “stretch” may have created a different phenotype in the infant lungs which was not equivalent to simple hyperoxia exposure in the mouse tissues. Additionally, the human tissues were assessed by immunofluorescence and subject to subcellular expression and quenching and the mouse tissues were measured by western blot. Furthermore, the current studies did not directly investigate ABPs in L-MSCs however we speculate that the changes in ABP homeostasis is relevant to changes in L-MSCs phenotypes during lung airway injury repair.

## Conclusions

**Fig. 6:**
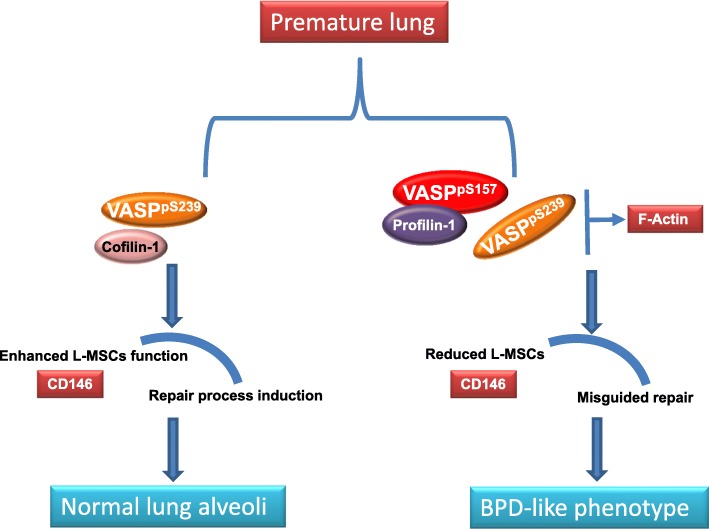
Schematic representation of hypothesis. Hyperoxia exposure decreases VASP expression and phosphorylation disrupting actin dynamics in human tissues. Early responses include increases in cofilin1 in attempts to initiate repair. In later stages of injury and dysregulated repair, we observe decreases in VASP phosphorylation and profilin1 which are indicative of impaired repair. L-MSCs play a significant role in repair and proliferate in response to injury stimuli. Early increase in CD146 is indicative of enhanced L-MSCs proliferation and homing in mouse lungs, while at later stages decreased CD146 in human autopsy tissue is suggestive injury beyond repair. We speculated that disruptions in ABP dynamics may be due to interventions or morbidities other than hyperoxia alone in humans with BPD

These studies indicate that ABPs are affected by hyperoxia exposure during development and suggest that the alterations in VASP throughout development and/or its interaction with other ABPs are likely to contribute to diminished repair processes and may also contribute to decreased lung growth and alveolarization (Fig. 6). Further investigations are warranted to specifically evaluate alternative ABPs and their precise role in mediating lung injury and repair in the developing lung.

## Abbreviations

ABPs: Actin binding proteins
BPD: Bronchopulmonary dysplasia
L-MSCs: Lung mesenchymal stem cells
O_2_: Oxygen
RA: Room air
VASP: Vasodilator-stimulated phosphoprotein
VASP^pS157^: VASP phosphorylation at serine 157
VASP^pS239^: VASP phosphorylation at serine 239
